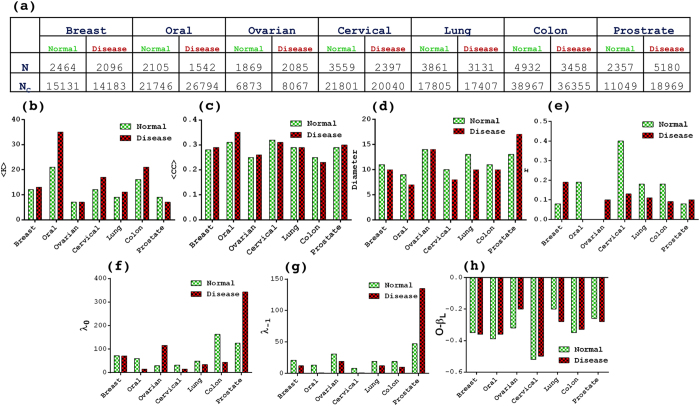# Corrigendum: Understanding cancer complexome using networks, spectral graph theory and multilayer framework

**DOI:** 10.1038/srep46646

**Published:** 2017-05-03

**Authors:** Aparna Rai, Priodyuti Pradhan, Jyothi Nagraj, K. Lohitesh, Rajdeep Chowdhury, Sarika Jalan

Scientific Reports 7: Article number: 41676; 10.1038/srep41676 published online: 02 03 2017; updated: 05 03 2017.

This Article contains an error in Figure 1, where the graph for Figure 1d is a duplicate of Figure 1b. The correct Figure 1 appears below.

## Figures and Tables

**Figure 1:**