# Supplementary material for: Understanding cancer complexome using networks, spectral graph theory and multilayer framework
Source: Sci Rep. 2017 Feb 3;7:41676. doi: 10.1038/srep41676 (PMC5290734; doi:10.1038/srep41676)
Supplement: Supplementary Information [file srep41676-s1.pdf]

Supplementary File

Understanding cancer complexome using networks, spectral graph theory and multilayer framework

Aparna Rai, Priodyuti Pradhan, Jyothi Nagraj, K. Lohitesh, Rajdeep Chowdhury, Sarika Jalan*

The inability in treating various cancers even after huge amount of investements demands development of tools from other branches of science and engineering than the core biology to understand the complexity of the disease. Here, we implement the network theory to compare the normal and disease tissues. The method implicates the deviation of cancer system from a random model. Also, the novel multilayer approach of these cancers along with the weak ties analysis: a tool used to study social systems, enables us to find proteins important for occurance of cancers which may be potential drug targets as well as may be useful to develop the concept of single drug therapy for various diseases.

1 Material and methods

Data collection and network construction: We retrieve the proteins of particular cancer by searching appropriate keywords of that cancer (e.g. for breast cancer normal breast cell, cancer breast cell/tissue etc are used). Thereafter, we elaborate on the other sources (cell-lines) from where we collect the proteins. The various cell-line databases used to obtain the list of proteins for different cancers are HMEC and MCF-7 cell line for breast normal and disease respectively, Cervical cancer database (CCDB) (http://crdd.osdd.net/raghava/ccdb/) for cervical cancer, ACTREC Oral Cancer Database and Head and Neck Oral Cancer Database (HNOCDB) for oral cancer, ATCC cell line database and Cancer Cell Line Encyclopedia for all considered cancers. For interactions we use STRING database. STRING database retrieves us the curated and experimentally verified protein-protein interactions that are direct (physical), indirect (functional) or both for a given list of proteins. The protein-protein interaction data of all the seven cancers for normal and disease states in the form of adjacency list can be found in Ref. 89 of the manuscript.

2 Structural and spectral properties

We determine various structural properties of all the seven cancers for the normal and the disease states and perform the comparitive analysis with their corressponding random networks constructed with the same average degree and probability $\langle k \rangle / N$ (Table. S1 and Table. S2).

Network	N	N^C	$\langle k \rangle$	$\langle CC \rangle$	r	r^{ER}	r^{conf}	$\langle CC \rangle^{conf}$	$O-\beta_L$
Breast$_{Normal}$	2464	15131	12	0.28	0.08	0	-0.05	0.03	-0.35
Breast$_{Disease}$	2096	14183	13	0.29	0.19	0	-0.04	0.03	-0.36
Oral$_{Normal}$	2105	21746	21	0.31	0.19	0	-0.04	0.06	-0.39
Oral$_{Disease}$	1542	26794	35	0.35	-0.03	0	-0.06	0.11	-0.36
Ovarian$_{Normal}$	1869	6873	7	0.25	-0.01	0	-0.05	0.03	-0.32
Ovarian$_{Disease}$	2085	8067	7	0.26	0.10	0	-0.04	0.02	-0.20
Cervical$_{Normal}$	3559	21801	12	0.32	0.40	0	-0.04	0.02	-0.52
Cervical$_{Disease}$	2397	20040	17	0.31	0.13	0	-0.04	0.03	-0.50
Lung$_{Normal}$	3861	17805	9	0.29	0.18	0	-0.05	0.01	-0.20
Lung$_{Disease}$	3131	17407	11	0.29	0.11	0	-0.04	0.02	-0.28
Colon$_{Normal}$	4932	38967	16	0.25	0.18	0	-0.04	0.03	-0.35
Colon$_{Disease}$	3458	36355	21	0.23	0.09	0	-0.05	0.05	-0.33
Prostate$_{Normal}$	2357	11049	9	0.29	0.08	0	-0.04	0.02	-0.26
Prostate$_{Disease}$	5180	18969	7	0.30	0.10	0	-0.04	0.01	-0.28

Table S1: The total number of proteins N and connections N_C, the average degree $\langle k \rangle$, average clustering coefficient $\langle CC \rangle$, assortativity value r for real and its corressponding configuration model, for the normal and disease networks.

2.1 Degree distribution

The degree distribution shows power law behavior for all the fourteen networks. Many network studies have reported an absence of the perfect power law for the overall range of the degree. Various real systems show a power law in the central part of data only and deviation from it in the small or the large scale. Several models have been used to explain the origin of the two power laws found in many systems. The models include, the geometric Brownian motion model, the preferential attachment model and the generalized model of the creation of new links between old nodes which increases with evolution time [1]. These models suggest that the evolution of a network is characterized by two parts, i.e., i) a leading ingredient of a network and ii) fluctuations within existing connections between nodes, being one of the reasons to lead the double power law nature of degree distribution. Here, the power law nature of the PPI networks implicates that the robustness of a network is maintained not only by the acquisition of new interactions by the hub proteins but also by the contribution of new or altered interactions within existing proteins for ease of pathway processes [2] which might have arisen due to the presence of internal physiological and external environmental factors during the evolution [3].

Network	N_{LCC}	N^C_{LCC}	D	λ_0 (%)	λ_{-1} (%)	D^{ER}	D^{conf}	λ_0^{conf} (%)	λ_{-1}^{conf} (%)
Breast_N	2443	15120	11	72 (2.9)	21 (0.8)	4	7	68 (2.8)	0
Breast_D	2046	14150	10	71 (3.5)	12 (0.5)	4	7	79 (3.9)	0
Oral_N	2105	21746	9	60 (2.8)	13 (0.6)	2	6	36 (1.7)	0
Oral_D	1542	26794	7	15 (1.0)	1 (0.0)	3	5	3 (0.2)	0
Ovarian_N	1748	6795	14	129 (7.4)	31 (1.7)	5	8	241 (13.8)	0
Ovarian_D	2022	8034	14	116 (5.7)	19 (0.9)	5	8	171 (8.5)	0
Cervical_N	693	4349	10	32 (5.0)	8 (1.3)	4	6	37 (6.1)	0
Cervical_D	792	6676	8	15 (1.8)	1 (0.1)	4	6	12 (1.4)	0
Lung_N	1256	5917	13	49 (3.6)	19 (1.5)	5	7	71 (4.3)	0
Lung_D	1026	5671	10	34 (2.2)	12 (1.4)	4	6	48 (2.8)	0
Colon_N	4849	38967	11	163 (3.3)	19 (0.3)	4	7	327 (6.7)	0
Colon_D	3423	36355	10	44 (1.3)	10 (0.2)	4	6	65 (1.9)	0
Prostate_N	2304	11020	13	125 (5.4)	47 (2.0)	5	8	133 (5.8)	0
Prostate_D	4938	18802	17	343 (6.9)	135 (2.7)	7	10	378 (7.6)	0

Table S2: Statistical properties of average over all the connected components for all the normal and disease datasets.

2.2 Degree- clustering coefficient Correlation

The overall Degree- clustering coefficient ($k - CC$) correlation for all the fourteen networks is negative as found for other biological systems [4]. However the graph of normal datasets indicate a deviation from this correlation pattern. It consists of a part of interactions yielding an overall negative $k - CC$ correlation, but some part of interactions is random reflected in absence of any $k - CC$ correlations. This inturn depicts that the disease datasets have an organized pattern [5] whereas, normal datasets deviating from any correlation and may be considered more random. This qualitative comparison is done on the basis of their random pattern, as sufficient amount of randomness is an essential ingredient for the functioning of the system [6]. The analysis infers that the lack of minimum amount of randomness in the network might be a cause of changes from the normal to the disease state by affecting the functional unit of the system (cell) through mutation and alterations in the interactions of proteins.

2.3 Eigenvalues distribution

The eigenvalue distribution of (a) all the disease and (b) normal networks show peak at zero eigenvalues. Overall both the states have triangular distribution as observed for other complex systems (Fig. S3).

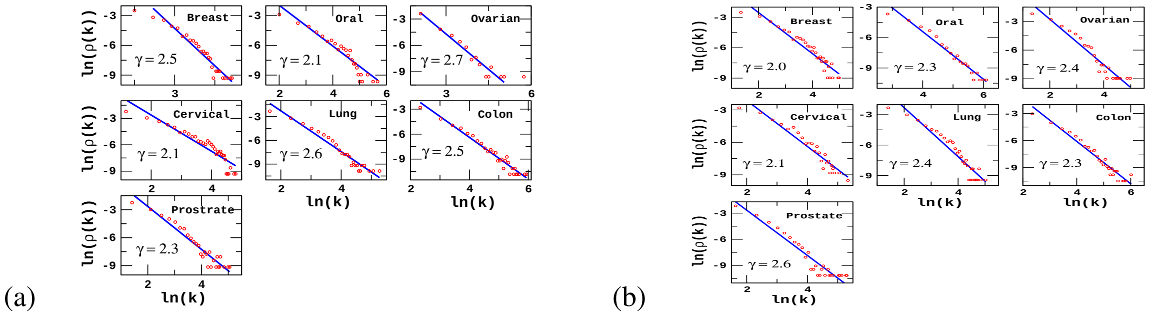

Figure S1: **Degree Distribution.** The degree distribution of all the networks follow power law behavior. Note that the fitting of the power law in not perfect and there are datapoints which do not fit the power law thus, shows double power law in many of the datasets.

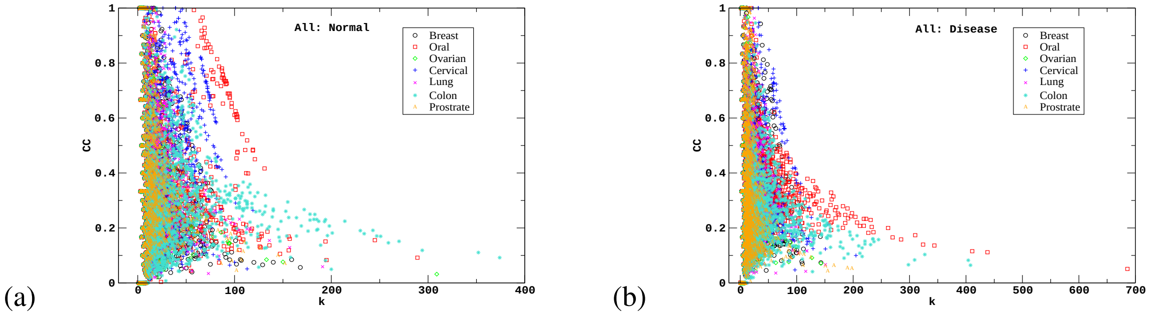

Figure S2: $k - CC$ **Distribution.** The $k - CC$ distribution of all the normal and disease possess overall negetive correlation.

2.4 Duplicated nodes

The duplicated nodes of all the disease datasets have different number of nodes which accounts for partial and complete duplications. Although there are few nodes which are same in some diseases. A screen shot of few duplicates are hereby enlisted (Fig. S4). The list of all the complete duplicate nodes for all the fourteen networks is given the Ref. 89 of the manuscript.

3 Common proteins

We enlist common proteins in all the normal and disease networks. What stands noteworthy is that, 19 proteins appear common in all the normal datasets and 71 in the disease ones. 8 proteins appear common in all the normal and disease datasets. The striking part which can be noted here is that among all the normal networks there are very less proteins which turns out to be common as compared to all the disease networks i.e. the disease networks have more common nodes among themselves.

Network	Hub Proteins
Breast$_N$	EGFR, CDK1, RAC1, CDH1, STX7
Breast$_D$	CAT, APC2
Oral$_N$	ALB, UBA52, TP53, YWHAZ
Oral$_D$	UBC, TSPO, ALB, AKT1, TP53
Ovarian$_N$	UBC, AKT1, TP53
Ovarian$_D$	EGFR, VEGFA, TP53, AKT1
Cervical$_N$	CDK1, SUMO2, BRCA1, SUMO1
Cervical$_D$	TP53, CDK1, AKT1
Lung$_N$	UBC, ALB, VEGFA
Lung$_D$	UBC, ALB, AKT1, TP53
Colon$_N$	TP53, AKT1, ABC27, ALB
Colon$_D$	TP53, AKT1, ALB
Prostate$_N$	ESR1, VEGFA, HRAS
Prostate$_D$	AKT1, TP53, ESR1, UBC, PTPRM

Table S3: **Hub Proteins.** The table depicts the significantly high degree nodes in all the fourteen networks.

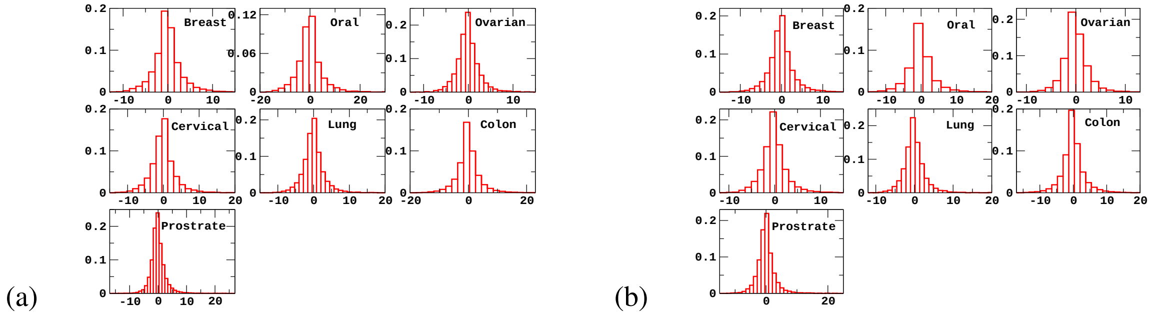

Figure S3: **Eigenvalues Distribution.** The eigenvalue distribution of (a) all the disease and (b) normal networks is triangular in shape.

The values of common proteins are enlisted in Table. S4. These 63, 11 and 8 proteins are further looked for their interactions in their corresponding individual normal and disease networks. The common proteins in each of these networks have different degrees due to the non-common proteins and addition or deletion of certain proteins in different diseases and thus the percentage interactions of these proteins in each state is different (Table. S5).

3.1 IN and OUT connections

The 63 proteins inetractiong among themselves are the IN connetions. OUT connections are the number of interactions these 63 nodes have other than the interactions among themselves in the network. The

(a)

breast	oral	ovarian	cervical	lung	colon	prostrate
ABCD3	RNF112	AGK	HIST1H4E	NID2	SSFA2	CELA2A
MYLK3	MAGEA6	CS	HIST1H4K	SP2	CEACAM3	FAM83D
KLK5	ZNF598	GTF3C4	HIST1H4J	IQSEC1	RNF115	PCSK2
TKT	EPS8L2	GPRASP1	HIST1H4I	FBXL12	FARP1	RNF115
RAB10	FAM49B	XPO4	HIST1H4D	RLF	ESM1	ZC3H14
VCP	TMEM109	CCDC115	C11orf58	MAGEA8	SLC39A8	IRX4
DNMT1	SSX7	CUEDC2	SIPA1	POU3F3	FOXP4	PCSK1
NSMCE2	SSX1	MOBKL1B	ZFP91	MTRR	LCORL	SP2
PTPN13	C20orf3	PGAM5	CRIP2	XPNPEP3	PDGFRL	SLC6A16
SORD	EPS8L1	AKR1C2	NRM29	FOXP4	FERD3L	TEK
LGALS1	C17orf81	GAS1	ASCC2	SPDEF	RHD	IQSEC1
LPCAT2	SLC25A13	EIF2B3	SHKBP1	PHLDB2	SMAP2	BACH2
LNPEP	SLC25A12	MLF2	HIST1H2AM	ANKRD13D	ASPHD1	KCNIP4
PUF60	ATP1B3	ALCAM	HIST1H2AL	CUL9	ZNF155	GRHL1
SLC39A10	PITPNB	CIB1	HIST1H2AI	BRD9	SLC30A6	ASRGL1
TRERF1	METTL7A	SLC16A3	HIST1H2AK	PTPN14	VASH1	CCDC110
BMP1	NAV3	A1BG	TKTL1	TAX1BP3	CEACAM7	HPSE2
ACSL4	FAM5C	RPUSD2	SEMG2	KANK2	OTOP2	DPT
GRB2		SLC3A2	GRM4	SLC39A12	KIAA0182	ERBB2IP
C21orf29		LRCH4	HIST2H4B	ARHGAP29	SORCS1	CHP2
CAP1		EIF4EBP1	TOMM34	TWF2	BAT2L1	TIMM50
PSME3		SRI	PHGDH	AKAP12	KCNK15	XTP3TPATP1
TDGF1		LYPD3	INPP5E	ARHGAP5	SLC35B2	NAA11
PRPF19		SPINK1	NRM	BTG1	NDRG2	PIK3C2B
TRAF4		TAX1BP3	SH2D2A	MOCOS	ENSG000002	POU3F3
CUX1		HSDL2	PYCR2	C18orf25	SCAF1	DLG5
ACP1		KDSR	CRNN	ENSG000001	CSRNP3	GUK1
RHOA		RASSF4	ETHE1	ADRA1A	ZNF25	ESRP2
AFAP1		EMILIN2	UBXN6	STARD3	ZNF22	ESRP1
FARP1		AP4M1	DTX3L	DCUN1D1	ENSG000002	NID1
MYO5C		SDF4	SMCHD1	ZNF839	AGER	THOC1

(b)

breast_nor	oral	ovarian	cervical	lung	colon	prostrate
AGK	RNF111	C13orf15	HIST1H4K	NID2	REM1	AGK
RNF115	TAS2R14	ZNF44	HIST1H4J	TRAPPC9	SSFA2	FTMT
TRAPPC8	TMPRSS11D	OPA1	HIST1H4I	SLC46A1	TCOF1	MAZ
ATP2A3	TMPRSS11E	MYO3A	HIST1H4D	PHLDA3	TRAPPC4	MAL
ERICH1	PGLYRP3	SHROOM3	ATXN2L	HRH1	ATP2A3	KCNJ8
MAGEA6	PGLYRP4	PELI1	TRERF1	SESN1	GTF3C2	PHLDA2
EIF4E2	SCMH1	SH2B1	RAB11FIP1	SULT1A4	KIF17	PRSS50
DBI	PAX8	BIN2	CPEB3	BFAR	TP53I13	AIG1
FABP7	MYO1D	ASTL	MAGEA1	SIRPG	ENSG000002	ILDR1
TNS4	ALDH1A3	IL31RA	YLPM1	RAB2B	TMPRSS11A	ARL11
GAS8	PCYOX1	RFX3	ESRP2	SMOX	ENSG000002	MID1
NBPF15	TAX1BP3	TMEM49	FNBP1L	CALU	SENP2	ATG4A
CANT1	RNF43	PINK1	GADD45GIP1	XPNPEP1	ENSG000002	RGS3
WDR69	DDIT4L	PIGY	TNS3	XPNPEP2	PGLYRP4	ABTB2
BRDT	HIST1H2AJ	CLIC4	PCIF1	PLCH1	PCDH17	CALML3
ENSG000002	HIST1H2AD	KIAA1191	GSDMB	PDGFRL	ALKBH2	LGR4
MBOAT7	TAS2R13	SVIP	MTPAP	DLST	ALKBH3	SH3D20
BCL2L12	SENP6	ZNF3	CARHSP1	KTN1	SYTL2	SLCO1A2
NISCH	TAS2R8	MYH7B	UTRN	TCEB3	RGS5	CANT1
ADRA1A	TAS2R9	AGR2	C8orf30B	PRDM5	MRPL38	UXT
MYST4	TAS2R4	TOPORS	C8orf30A	FLCN	LSAMP	PERP
TTC3	TAS2R5	PER1	NDRG2	DUS2L	YTHDF1	LPIN2
SPDEF	ITLN2	TMEM209	USP42	PRDM1	AZGP1	PNRC2
SORT1	CCDC8	ATXN10	SLC39A10	LAIR1	731682	FAM48A
MAP3K6	TAS2R20	FBXO31	PTPN9	NDRG2	KIAA1468	CHD1L
SLC1A4	DNASE2B	FSD1L	MYO1D	MOG	CLDN19	PPM1M
EEPD1	STXBP4	PLIN2	SLC6A15	FSTL1	MLF2	C4B-1
JAKMIP1	RAB40AL	NUCB2	ATXN10	GRSF1	C1orf31	ADAMTS18
NOMO2	FREM2	G6PC3	KANK2	ARHGAP29	CRIP1	SLC6A19
SLC1A5	SPRR2E	RCN1	ALPI	COL6A1	FGF14	UGT1A10
EXOSC2	SPRR2F	RNF43	MTBP	DEDD2	RELT	SLC39A10
KRIT1	KRT81	FAM120B	ARHGAP21	KIR3DL1	ENSG000001	PTOV1
KIAA1009	KRT86	REPS1	FAM76B	PLIN2	CLCN1	IL22RA2

Figure S4: The left panel depicts few duplicates in disease states, while right panel shows a screen shot of the duplicates in the normal dataset. The number duplicate nodes differ in all the datasets.

Common in both	All disease	All normal
8	63	11

Table S4: **Common proteins.** The table depicts all the common proteins among the normal and disease datasets and uniquely common is all disease and normal states.

total number of interactions is denoted by $Total$ which implies to the sum of the IN and OUT connections of these 63 nodes in the network. The Table. S6 depicts the above explained terms

The human protein atlas [7] and KEGG pathway [8, 9] analysis of 63 proteins reveals involvement of these proteins in similar type of pathways also known to be forming modules (Table. S7 and Fig. S5 (Figure obtained from KEGG database).

Role of pathways in Cancer (where 63 common proteins are involved)

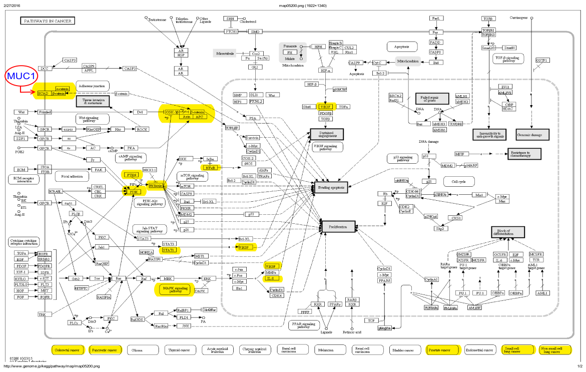

Figure S5: **KEGG pathway analysis.** The KEGG pathway analysis depicting the role of 63 proteins. Source: KEGG database [8]

**PPI Network**	$I_{DC}(\%)$	$I_{NC}(\%)$	$I_{AC}(\%)$
Breast	30	21	8
Oral	66	54	13
Ovarian	30	17	9
Cervical	41	31	6
Lung	28	12	5
Colon	42	22	11
Prostate	18	41	11

Table S5: **Number of interactions common proteins share in individual PPI networks** I_{DC} denotes the neighbors of the 63 nodes. I_{NC} denotes the percentage of nodes covered by the 11 nodes and I_{AC} shows the interacting partners in their corresponding disease networks.

Network	P_I	IN	OUT	Total
Breast	616	13	914	927
Oral	1025	274	4500	4774
Ovarian	980	84	1154	1238
Cervical	1439	105	2252	2357
Lung	882	54	1502	1556
Colon	620	182	3937	4119
Prostate	929	87	1555	1642

Table S6: **Neighbors of the 63 proteins common to all the seven cancers.** P_I denotes the neighbors of the 63 nodes (excluding the 63). The 63 proteins inetractiong among themselves are the IN connetions, whereas OUT connections imply to the number of interactions other than the 63 nodes in the network. The total number of interactions ($IN + OUT$) is denoted by $Total$.

4 Network Theory revealing important proteins

Network theory discussed above provided valuable information on proteins showing high betweeness and low degree (HBLD). We performed a literature survey followed by pathway analysis through STRING software to determine the probable functions and interacting partners of these proteins respectively [10]. We found that these proteins are well implicated in cancers (Table. S8). Based on their functional aspects we characterized the six proteins into two categories:- **"A as sensors"**, which included four proteins and **"B as effectors"** comprising of two proteins (Figure 5 of the manuscript). The proteins marked under "A" category are primarily upstream components of intra-cellular signaling cascades, expression of which is altered under cellular stress or upon change in cellular internal micro-environment eventually leading to activation of downstream genes triggering cancer or its progression. The proteins under "B" category are downstream effectors which are often implicated but not exclusive to cancer. The interacting partners of the proteins were selected based on probabilistic confidence score. They included both direct and indirect ones; direct partners are those that show physical binding or have sites for phosphorylation,

while, indirect partners are activated or affected indirectly by them. The associations in STRING are based on high throughput experimental data, deep search of the databases and predictions based on genomic context analysis. The cut off for interaction mapping and selection was high experimental and biochemical data of > 0.50 score and a score indicating evidence of specific actions > 0.50. Based on results from interaction analysis a schematic of interacting molecules with category A group of proteins was created. The interaction pattern has significant and specific correlation with proteins involved in proliferation and migration of cancerous cells; interactome map with proteins from B category, showed many interacting partners with functions implicated in cancers. This was an expected outcome since they are downstream effectors of multiple signaling cascades. For further elaborative studies "B" category proteins were not considered. Finally, utilizing and incorporating information of interacting partners, by KEGG pathway analysis, we filtered out probable pathways through which these proteins can affect cancers (Figure 6(A) in the manuscript). Given below is a description of identified functions of category "A" proteins and its association with cancer:

The detail functions are as follows:

1 . MUC1: Interaction of Mucin1 with EGFR can activate a myriad of signaling pathways one of which is STAT1 [11–15]. The later along with STAT3 is well known to activate VEGF (vascular endothelial growth factor) which sustains and augments angiogenesis in cancer cells [12, 16, 17]. The second important interaction of MUC1 which is well studied is with the ErbB heterodimers leading to activation of Ras-responsive genes. The direct effect of this interaction is activation of MAPKs enhancing progression of cancer. MUC1 is also reported in literature to hinder the inhibitory interaction of E-cadherin with β catenin, which leads to increased availability of -catenin inside the nucleus that activate transcription factors responsible for progression of cancer [18]. A family of tyrosine kinases called protein tyrosine kinases which function as proto-oncogene are also reported to be activated by MUC1; these include SRC, LYN, LCK proto-oncogenes [19]. The schematic representation of the pathways involved can be found in Fig. S6.

2 . SOD2: SOD2 is a well-studied molecule playing regulatory roles in cancers, however it is controversially discussed in literature [20]. SOD2 is reported to modulate IKK expression activating NF-B and thereby facilitating proliferation and metastasis [21]. The Forkhead transcription factors of the O class are known in detoxification of ROS through induction of SOD2 [22, 23]. However, FOXOs are implicated as tumor suppressors and are important targets of PI3K pathway which regulates a cascade of other

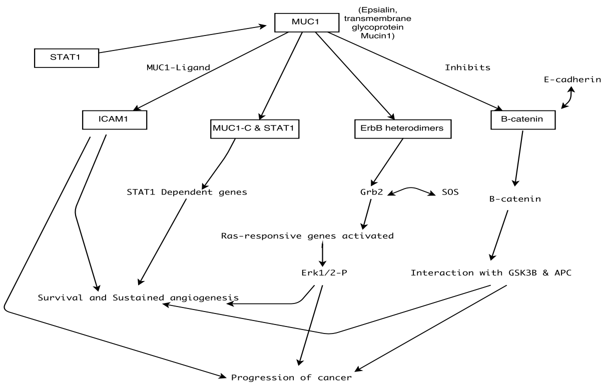

Figure S6: Pictorial view of the role of MUC1 protein in a cell.

signaling pathways in cancer cells [24]. Recent reports have highlighted the modulation of SOD2 by a member of NF-B by regulating FOXO3 which thereby proves their inter-relation [25, 26]. The pictrial representation for the role of protein is illustrated in Fig. S7.

3 . HSPA4 and HSPA5: The heat shock family of proteins though present in normal cells are expressed at high levels under temperature stress and in cancer cells under altered pH and oxygen deprivation. Both HSPA4 and A5 are reported to interact with HSP90 which is a highly conserved chaperone. It is known to facilitate the maturation of wide range of proteins some of them being oncogenes. In fact it is said that the unpredictable activities of oncogenes are enabled by HSP90 directly [27]. Interactions of HSPA4 and A5 with molecular chaperone regulator BAG proteins are also reported. BAG is strongly associated with the malignant behavior of tumors and known to interact with potent transcriptional regulator-HIF1alpha, steroid hormone receptors and anti-apoptotic factors like Bcl2 positively regulating their function [28]. Overall, in a wide variety of cancers, HSPA4 and A5 are correlated with proliferation, cell migration, apoptosis and drug sensitivity enabling typical cancerous characteristics [29–33].

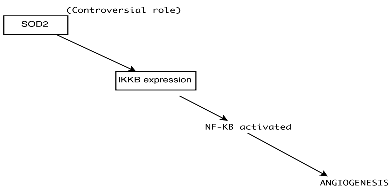

Figure S7: Schematic diagram of the role of SOD2 protein and pathways associated.

5 miRNAs regulating proteins with high betweeness: An important application driven from Network theory

5.1 Motivation behind studying miRNAs among many others

In the era of personal genomics, there is a growing number of evidence that supports that non-coding regions regulate gene expression and RNA processing. Also, with the advent of ENCODE (Encyclopedia of DNA elements) project in 2005, attempts were made to annotate all elements of the human genome. Since then the non-coding genomic data has become more and more comprehensive and databases have been created that record and predict their role. Amongst various non-coding regions, miRNAs are the most studied. We chose miRNAs for our study because in the present era, genetic marker based studies for any disease are incomplete without supporting information of non-coding RNAs that can potentially modulate gene expression. Also, miRNA databases are enriched with both in silico prediction and experimentally validated datas providing valueable information for any related study. Hence, miRNA based regulation of the critical genes obtained from our study is performed and compared with existing results to provide a holistic information of genetic regulation in the cancer models selected.

MicroRNAs (miRNAs) are a class of short non-coding RNAs with posttranscriptional regulatory func-

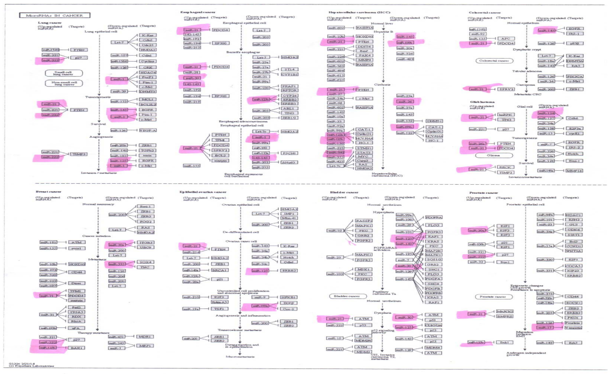

Figure S8: The KEGG analysis of the miRNAs showing regulation of proteins with particular miRNA in the cancers considered here.

tions. Recent studies have shown that the expression of miRNAs is de-regulated in cancer progression, tumor invasion, metastasis, and subsequent chemoresistance [34, 35]. There are complementarity between specific regions of mRNAs and miRNA that ultimately leads to inhibition of gene function. In this study, through in silico analysis we initially shortlisted miRNAs that has high binding affinity with the "A" group mRNAs. Thereafter, we checked whether the selected miRNAs can also potentially regulate the interacting partners of the "A" group proteins found from interactome studies. Importantly, a single miRNA can bind and regulate the function of multiple mRNAs. We used the Sanger miRNA registry-miRTarBase for the in silico miRNA prediction analysis of target genes [36]. Following the prediction of miRNAs with high binding affinity to genes implicated in network analysis, we analyzed the reported expression pattern of these miRNAs in various cancers. Our study provides a wholesome picture of proteins over-expressed in cancer, their interacting partners, the pathways they may be involved in and information on epigenetic regulators involved in controlling expression of the above proteins. Some of the striking observations from miRNA prediction and analysis is given in Table. S9 and Table. S10.

Reports exist in literature showing binding motifs of the microRNA-miR-125b in MUC1-3UTR, negatively regulating the gene [37]. Our miRNA analysis also predicted miR-125b as a probable miRNA regulating MUC-1. Interestingly, KEGG analysis of various miRNAs de-regulated in cancer highlights miR125 as a family of miRNA down-regulated in various cancers. This provides strong indication to the cause towards over-expression of MUC-1 in cancer cells. Additionally, the targets of miR125 depicted in KEGG pathway includes some proteins like, EGFR, ERBB2, P53, CDKN2A which were found to interact with MUC1 through interactome analysis described above. This commonality provides interesting clues towards regulation of not only a HBLD gene but also multiple associated genes implicated in cancer to be regulated by the same family of miRNA. In a similar manner, miR145 was also pre-

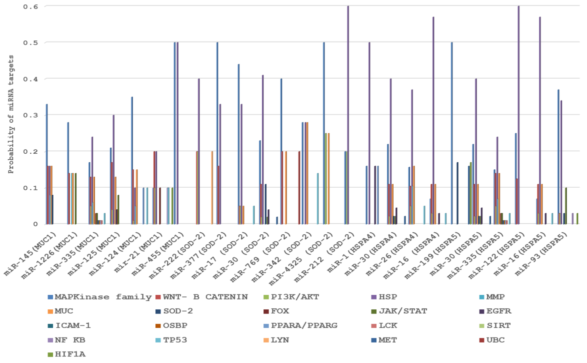

Figure S9: Graphical view of the probabilistic distribution of different targets regulated by miRNAs regulating sensors.

dicted as a miRNA with potential binding affinity for 3-UTR of MUC1 and this miRNA is suppressed in lung, esophageal, hepatocellular, colorectal and bladder cancer [38]. This miRNA also targets genes like EGFR, FGFR3 and PKC which have well established role in tumor invasion and metastasis. Interestingly, we found miRNAs which are implicated to regulate "A" group proteins being up-regulated in cancers (Fig. S8). These include miR-21, miR-222 and miR-26 family of miRNAs. However, they are reported to down-regulate tumor suppressor genes like PTEN, BMPRII, RECK, TIMP3, BCL2, PDCD4, TPMI thereby promoting metastasis and survival (Table. S9). To have a complete idea about miRNA-mediated regulation, we calculated the probabilistic distribution of proteins regulated by a given miRNA which also controls the expression of Group "A" proteins. The MAPKinase family was found to be a highly probable target (Fig. S9). The implication being that proteins of this signaling pathway is highly important in these 6 types of cancers and it can be chosen as a suitable target to be looked upon after miRNA inhibition. This list was followed by the HSPs, Wnt/B-catenin, PI3K/Akt, Mucin family, based on calculating the probability scores alone (Table. S10). In conclusion, the data indicates the merits of using Network theory to predict plausible nodes/proteins regulating a range of downstream targets. However, experimental validation is essential for a concrete conclusion.

[redacted]

Sr.	**Protein target**	**miRNA**	**Validated targets**	**Potential targets**
1.	MUC1	miR-145	SOD2; STAT1; EGFR; MAP2K6, ETS1; MMP12, MMP14; CTNND1; MYC	MMP1, MUC19, MUC4; SOD2; DNAJC28, DNAJC10; ERBB4, CTNNB1P1; MAP3K3, MAP2K4, MAP3K11, MAP4K2
		miR-1226		CTNNB1, MAP3K7, MAP2K6; MMP17; GALNT2; STAT5B; PIP5KIA

		miR-335	MAPK1	WNT9A, WNT7B, GRB7, GRB10, SOS2, WNT3, WNT10B, CTNNB1; MAPK4, MAP3K8, MAPK14, MAP3K7, MAP2, MAP2K5, MAP1LC35,RASGRP1,RASGRP3, RASA2;HSPA1B, HSPA1L, HSP90B1, HSPA1A, HSPA5, HSP90AB1, HSPA6; DNAJB14, DNAJB9, DNAJC13,DNAJC21, DNAJA1, DNAJC3, DNAJC54; FOXO1, FOXO3; ICAM-1; EGFR-1; OSBP; PPARA; LCK; JAK3,JAK2; TP53AIP1, TP53INP1; PIK3CA, PIK3C2B, PIK3C2A; MUC3A, MUC4, MUC5, MUC15 MUC5AC, MUC6, GALNT16, GALNT25; MMP20,MMP14,MMP2,MMP17
		miR-125	TP53, TP53INP1; ERBB2, ERBB3; AKT1; MMP11; MAPK14; ICAM1; EGFR	STAT2, STAT3; MAPK8, MAPK1, MAPK6, MAP3K9, MAPK14; WNT3, WNT7B, GRB2; SOD2; PIK3C2A; GALNT18, GALNT4, GALNT7; FOXO1; HSPA4, HSPA1B, HSPA4, DNAJC24, DNAJB4, DNAJB9, DNAJB4
		miR-124	WNT4; STAT3; MAPK14; SIRT1; PIK3CA	MAP3K8, MAPK2, MAPK14, MAPKAPK3, MAP2K3, MAP3K3, MAP3K4; SOS2, WNT5B, ERBB2; AKT2, AKT3; PPARA; TP53INP1; MUC1, GALNT7, GALNT4; DNAJC25, DNAJC1; MMP19
		miR-21	EGFR; ICAM1; PPARA; MMP2; STAT3; AKT2; MAP2K3, MYC; ErbB2	TP53; NFKB1; MAP3K1; FOXO3; WNT1, WNT5A; PIK3C2A; DNAJC16, DNAJC10; HIF1A
		miR-455		HSP90AB1, DNAJC18; MAP3K9, MAP3K2;
2.	SOD2	miR-222	STAT5A; MMP1; FOXO3; ICAM1; TP53, TP53BP2;	LYN; AKT3; HSPA14, HSP19AA1; GRB10;
		miR-377		HSPA6, HSPA12B; MAP3K9, MAPKAPK5, MAPK8IP2; GSK3B
		miR-17	JAK1, STAT3; ICAM1; MAPK9, MAP3K12, MAP3K3; DNAJC27; HIF1A; MUC17; SOD2; MMP2; GALNT7; TP53INP1	PIK3CA; MUC21; RRAS2, MAP3K8, MAP3K9, MAPK14, MAP3K21, MAPK1, MAPKAPK5, MAP3K14; DNAJB4, DNAJB13, DNAJB9, DNAJC10, DNAJB6, DNAJC28; TP53; WNT10B

		miR-30	TP53; ESR1, ESR2; PIK3R2, PIK3CD; ERBB4; MUC17; WNT5A; HSPA4, HSPA5; NF-KB1A	MET; HSP90AA1, HSPA1B, HSPA6, HSPA1B, DNAJC2, DNAJB1, DNAJC3, DNAJA1, DNAJB14, DNAJC8, DNAJC24, DNAJA4, DNAJC2, DNAJB1, DNAJB4, DNAJC30, DNAJB9, DNAJC10; EGFR1, EGFR; WNT4, WNT5A, ERBB3, CTNNB1, WNT7B; MAPK1, MAP2K1, MAPK14, MAPKBP1, MAP2K3, MAPK8, MAPK1IP1L, MAPK4, MAP2K1, MYC; JAK1; PIK3C2B; GALNT1, GALNT10, GALNT6, GALNT7, GALNT1
		miR-769		UBC; CTNNA1; GALNT10; MAPK1, MAP3K5
		miR-342		DNAJC8, DNAJC9; MUC17; TP53INP2; GALNT10; MAPK10, MAPKBB1
		miR-4325		MAPK8, MAP2K4; GALNT3, PIP5K1C
		miR-212		MAP1LC3B; PIK3C2A; HSPA1B, HSPA4L; DNAJB4
3.	HSPA4	miR-1	SIRT1; PIK3CA; HSPA4	EGFR; DNAJB1, HSPA1A, DNAJC10; MAP4K2; PPARG;
		miR-30	TP53; ESR1, ESR2; PIK3R2, PIK3CD; ERBB4; MUC17; WNT5A; HSPA5; NF-KB1A	MET; SOD2; DNAJC2, DNAJB1, DNAJC3, DNAJA1, DNAJB14, DNAJC8, DNAJC24, DNAJA4, DNAJC2, DNAJB1, DNAJB4, DNAJC30, DNAJB9, DNAJC10; EGFR1, EGFR; WNT4, WNT5A, ERBB3, CTNNB1, WNT7B; MAPK1, MAP1B, MAP2K1, MAPK14, MAPKBP1, MAP2K3, MAPK8, MAPK1IP1L, MAPK4, MAP2K1, MYC; JAK1; HSP90AA1, HSPA1B, HSPA6, HSPA1B; PIK3C2B; GALNT1, GALNT10, GALNT6, GALNT7, GALNT1
		miR-26	MAP3K2; GSK3B; ESR1	DNAJC2, DNAJB4, DNAJA2, DNAJA3, HSPA1L, HSPB7, HSPA13; MMP10, MMP8; GRB7, WNT5A, TP53I3; AKT1; GALNT6, GALNT3, MUC7; MAP2K4, MAP3K12, MAP3K9
		miR-16	AKT3; WNT3A, WNT4; TP53; MAP7	HSPA1A, HSPA1B, HSP90B1, HSPA9, HSPA5, HSP90AA1, DNAJA1, DNAJA4, DNAJA2, DNAJB1, DNAJB4, DNAJC2, DNAJC9, DNAJC15, DNAJC10; GALNT7, GALNT3, GALNT1; EGFR; GRB2, GSK3B, WNT5A; TP53INP1; PIK3RL; MAP4K2, MAPKAPK2

4.	HSPA5	miR-199	IFKB; NF-KB1 MET; AKT1; WNT2, GSK3B, ERB2, ERB3; SIRT1; DNAJA4; HSPA5; MAPK1, MAPK9, MAPK8, MAPK14, MAP3K11	MAPK1, MAP3K4, MAP3K9; MET; HIF1A; SOD2
		miR-30	TP53; ESR1, ESR2; PIK3R2, PIK3CD; ERBB4; MUC17; WNT5A; HSPA4; NF-KB1A	MET; SOD2; HSPA1B, HSPA6, HSP90AA1, HSPA1B, DNAJC2, DNAJB1, DNAJC3, DNAJA1, DNAJB14, DNAJC8, DNAJC24, DNAJA4, DNAJC2, DNAJB1, DNAJB4, DNAJC30, DNAJB9, DNAJC10; EGFR1, EGFR; WNT4, WNT5A, ERBB3, CTNNB1, WNT7B; MAPK1, MAP2K1, MAPK14, MAP-KBP1, MAP2K3, MAPK8, MAPK1IP1L, MAPK4, MAP2K1, MYC; JAK1; PIK3C2B; GALNT1, GALNT10, GALNT6, GALNT7, GALNT1
		miR-335	MAPK1	WNT9A, WNT7B, GRB7, GRB10, SOS2, WNT3, WNT10B, CTNNB1; MAPK4, MAP3K8, MAPK14, MAP3K7, MAP2K5, MAP1LC35,RASGRP1,RASGRP3, RASA2; HSPA1B, HSPA1L, HSP90B1, HSPA1A, HSPA5, HSP90AB1, HSPA6, DNAJB14, DNAJB9, DNAJC13,DNAJC21, DNAJA1, DNAJC3, DNAJC54; FOXO1, FOXO3; ICAM-1; EGFR-1; OSBP; PPARA; LCK; JAK3,JAK2; TP53AIP1, TP53INP1; PIK3CA, PIK3C2B, PIK3C2A; MUC3A, MUC4, MUC5, MUC15 MUC5AC, MUC6, GALNT16, GALNT25; MMP20,MMP14,MMP2,MMP17;
		miR-122	MAPK2; AKT3; WNT1; GALNT10	MAPK1, MAP4K2; WNT2B; DNAJB1, DNAJC18, DNAJB13; HSPA5, HSPA4L;
		miR-16	AKT3; WNT3A, WNT4; TP53; MAP7	HSPA1A, HSPA1B, HSP90B1, HSPA9, HSPA5, HSP90AA1, DNAJA1, DNAJA4, DNAJA2, DNAJB1, DNAJB4, DNAJC2, DNAJC9, DNAJC15, DNAJC10; GALNT7, GALNT3, GALNT1; EGFR; GRB2, GSK3B, WNT5A; TP53INP1; PIK3RL; MAP4K2, MAPKAPK2

miR-93	TP53INP1; MAPK9; ICAM1; WNT2B	MAP7, MAPK2, MAP2K1, MAP3K13, MAP3K4, MAP3K3, MAP3K2, MAPK1, MAP3K14, MAPKAPK5, MYC; STAT3, STAT5B, JAK1; AKT1P; SOD2; NFKB1L1; HSPA5, HSP90AB1, HSPA8; DNAJB9, DNAJC27, DNAJB6, DNAJB13, DNAJC28, DNAJB4, DNAJC5, HIF1A, GRB2

Table S10: **List of validated and potential targets of the miRNAs regulating Group "A" proteins.** A probabilistic score of the family most likely to be regulated by the same miRNA is highlighted(Source: miRTarBase)

6 Other properties

6.1 Entropy

To understand in depth the fundamental differences and similarities between all the cancers, we further determine the entropy of all the networks. Entropy is defined as the disorderness in any system. The Von Newman entropy and shannon entropy are known to characterize the graph [54, 55] Here, we calculate the entropy for network attributes of the network such as degree (H_k), clusterring coefficient (H_{CC}), and eigenvalues of the laplacian matrix (H^L_λ), as defined in the methods section. We scale them for a better comparision by evaluating γ values classify all the cancers into superfamilies. The system is more complex if the value of γ is high.

The γ corressponding to the entropy due to the degree (γ^k) is shown in Table. S11, which reflects that the oral cancer dataset is more complex than its normal network as the value of γ in the disease is more than its normal state while, the ovarian cancer has normal dataset more complex than the disease. All other networks show almost equal γ values for both the states.

Similarly, γ corressponding to the entropy due to the clusterring coefficient (γ^{CC}) depicts that disease states of breast, oral, cervical and colon cancer have more γ than its normal. The disease states of these cancer being more complex may be due to the presence of more number of modules in the network. The datasets of ovarian, lung and prostate cancers have nearly equal γ values.

The γ corressponding to the entropy due to γ^L_λ enlists that except for the breast and the oral cancer all other diseases have equal value of γ. The oral cancer as mentioned above has cancer state more complex than the normal one whereas, for the breast cancer normal dataset is more complex than the disease one.

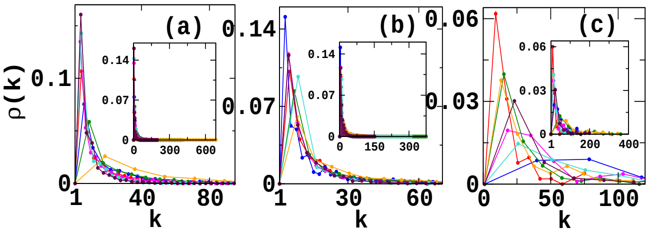

Figure S10: **Degree Distribution.** The degree distribution of (a) all the disease, (b) normal networks and (c) 63 nodes commonly present in all the disease networks show a single peak, all between $5 - 20$ deciphering no significant contribution of degree for all the common nodes. Different colors of circles depict seven cancers in (a, b and c) each.

Summarizing the above, we find that the disease state of oral cancer is found more complex than its normal counterpart for all the γ values. Also, for CC distribution, breast, oral, colon and cervical cancer states have similar behavior of complexity i.e. the complexity is found increasing from normal to disease state.

7 Properties of Common proteins

Further, we find that there are 63 proteins which are common (Table. S4) in all the disease. These proteins are further looked for their interactions in the individual disease network. The proteins in each of the disease networks have different degrees due to the non-common proteins and addition or deletion of certain proteins in different diseases.

7.1 Degree distribution

The degree distribution of all the normal and disease networks exhibit power law behavior (Fig. S10(a,b)), indicating the abundance of low degree nodes and presence of few hub nodes with structural as well as functional importance [56]. The degrees of the 63 common disease-associated proteins are also distributed in a power law fashion (Fig. S10(c)), indicating that the commoln subset of nodes also scale similar to the disease and normal networks with few high degree nodes. Interestingly, the 63 nodes common in all the disease networks aquire around 30-40 percent of the interactions in individual disease networks except oral and prostate cancer which have 71% and 19%, respectively (Table. S5).

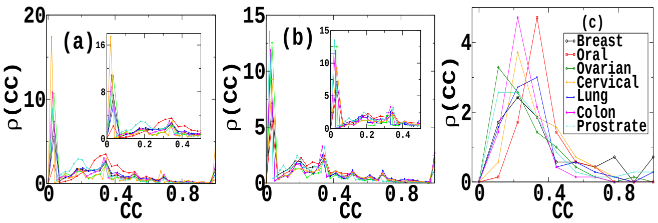

Figure S11: **CC distribution.** The CC distribution reveals the presence of two major peaks in all the (a) disease and (b) normal networks ($0.02 - 0.1$ and $0.2 - 0.4$). The nodes common in all the disease network (c) shows the peak between $0.2 - 0.4$ which comes under the second highest peak in the disease dataset.

7.2 CC distribution

The $\langle CC \rangle$ of all the disease and normal networks is very high.(Fig. S11(a, b)). The distribution of CC depicts two major peaks in the disease networks with the second highest peak lying in the regime 0.2-0.4.Interestingly, the peak of CC distribution for the 63 common disease proteins also lies in this regime (Fig. S11(c)) indicating the accountability of these proteins for the second major peak in the disease networks.

7.3 k-CC correlation

The k-CC correlation of all the disease and normal networks show overall negative correlation as also depicted by many other biological systems indicating the presence of hierarchical structure in the system [56]. The presence of hierarchical structrures is an indication of highly clustered neighborhoods consisting of sparsely connected nodes communicating through hubs [57]. The presence of modules to an extent may be responsible for the system to function properly and thus may help the patient prolong the process of system failure.

8 Functional Properties of 63 proteins

Sr.	**Protein name**	**Functions**
1.	HSPA5	Involved in the folding and assembly of proteins in the ER. As this protein interacts with many ER proteins, it may play a key role in monitoring protein transport through the cell; and in facilitating the assembly of multimeric protein complexes inside the ER. Inhibit apoptosis.
2.	HSPA4	Inhibit apoptosis, cellular response to stress.

3.	HSPA9	Tumor suppressor gene, overexpression closely associated with advanced tumor stages having implications for increased malignancy and aggressive behavior.
4.	MUC1	Inhibit apoptosis.: Play an essential role in forming protective mucous barriers on epithelial surfaces. Association of MUC1 with p53 in cancer results in inhibition of p53-mediated apoptosis and promotion of p53-mediated cell cycle arrest. These proteins also play a role in intracellular signaling. Overexpression of MUC1 is often associated with colon, breast, ovarian, lung and pancreatic cancers.
5.	TPM4	Involved in the contractile system.
6.	PRDX5	Antioxidant mechanism and signal transduction. Among its related pathways are Metabolism and Cellular Senescence.
7.	PRDX2	Might participate in the signaling cascades of growth factors and tumor necrosis factor-alpha by regulating the intracellular concentrations of H_2O_2.
8.	PRDX1	Involved in apoptosis related pathways.
9.	AKT1	Regulate many processes including metabolism, proliferation, cell survival, growth and angiogenesis.
10.	RPS3	DNA repair activity directed towards the mutagenic lesions. Higher levels of expression of this gene in colon adenocarcinomas and adenomatous polyps compared to adjacent normal colonic mucosa have been observed.
11.	MMP14	Specifically activate progelatinase A. May thus trigger invasion by tumor cells by activating progelatinase A on the tumor cell surface. Acts as a positive regulator of cell growth and migration via activation of MMP15.
12.	EPCAM	Involved in cell adhesion, cell-cell interaction. Oligomerization of this leads to regulation in cell adhesion and metastasis.
13.	AIFM1	Involved in regulation of caspase- independent apoptosis.
14.	NBN	Plays a critical role in the cellular response to DNA damage and the maintenance of chromosome integrity. The complex is involved in double-strand break (DSB) repair, DNA recombination, and maintenance of telomere integrity, cell cycle checkpoint control and meiosis.
15.	LMNA	Plays an important role in nuclear assembly, chromatin organization, nuclear membrane and telomere dynamics. Transcriptional regulation, mechanism of aging.
16.	MTUS1	Tumor suppressor and participates in AT2 signaling pathways. Cooperates with AGTR2 to inhibit ERK2 activation and cell proliferation.
17.	CTTN	Contributes to the organization of the actin cytoskeleton and cell structure. Plays a role in the regulation of cell migration. Involved in the invasiveness of cancer cells, and the formation of metastases.
18.	FN1	Fibronectins are involved in cell adhesion, cell motility, opsonization, wound healing, and maintenance of cell shape.
19.	BRIP1	Required for the maintenance of chromosomal stability. Acts late in the Fanconi anemia pathway, after FANCD2 ubiquitination. Involved in the repair of DNA double-strand breaks by homologous recombination in a manner that depends on its association with BRCA1. May be a target of germline cancer-inducing mutations. In some cancers also acts as tumor suppressor.

20.	ENO1	Role in glycolysis, plays a part in various processes such as growth control, hypoxia tolerance and allergic responses. May be involved in TGF-beta signaling. In multiple tumors promote cell proliferation, cancer invasion,
21.	PTK2	Involved in cellular adhesion and regulating cell migration and spreading, Role in Apoptosis. Cell cycle progression, cell proliferation.
22.	MSH2	Act as tumor suppressor gene and more specifically a caretaker gene that codes for a DNA mismatch repair in cancer. Very much prevalent in colorectal cancer.
23.	ARHGDIA	Play a role in cellular proliferation and differentiation. In glioma cells, inhibits cell migration and invasion.
24.	FGFR2	Acts as cell-surface receptor for fibroblast growth factors and plays an essential role in the regulation of cell proliferation, differentiation, migration and apoptosis, and in the regulation of embryonic development.
25.	NME1	Involved in cell proliferation, differentiation and development, signal transduction. Reported to participate in metastasis in some cancers.
26.	GNAS	Involved in the trans membrane signaling. Regulation in the expression is found in cell cycle and cell proliferation in few cancers.
27.	BIRC5	Also known as survivin. Multitasking protein, has dual roles in promoting cell proliferation and preventing apoptosis. For some cancers due to regulation, it is regarded as an oncogene. Some studies reveal that it might provide a new target for cancer therapy that would discriminate between transformed and normal cells. Survivin expression is also highly regulated by the cell cycle.
28.	MLH1	involved in the DNA mismatch repair. Decreased expression in various cancers.
29.	SOD2	Inhibits apoptosis, promotes angiogenesis. Overexpression of SOD2 has been linked to increased invasiveness of tumor metastasis. Has role in controlling ROS levels, which also involves it in ageing, cancer, and neurodegenerative disease.
30.	MAPK1	Involved in many pathways like proliferation, differentiation, transcription regulation and development.
31.	PYCARD	Functions as key mediator in apoptosis and inflammation. Involved in activation of the mitochondrial apoptotic pathway.
32.	PTEN	Tumor suppressor. Regulation of this protein leads to inhibition of apoptosis, hyperplasia and tumor formation.
33.	MMP10	Metastasis, also helps in degrading a wide range of extracellular molecules. Play a central role in cell proliferation, migration, differentiation, angiogenesis, apoptosis and host defences. Dysregulatoin of MMP10 has been implicated in many diseases including arthritis, chronic ulcers, encephalomyelitis and cancer.
34.	VEGFA	This Growth factor is active in angiogenesis, vasculogenesis and endothelial cell growth. Induces endothelial cell proliferation, promotes cell migration, inhibits apoptosis and induces permeabilization of blood vessels. Found important in many cancers.
35.	S100A8	calcium- and zinc-binding protein. Role in the regulation of inflammatory processes and immune response. Also, in regulation of a number of cellular processes such as cell cycle progression and differentiation.
36.	DAB2	Tumor suppressor gene in many cancers through methylation, Act as transcriptional regulator.

37.	CDH1	Involved in mechanisms regulating cell-cell adhesions, mobility and proliferation of epithelial cells. Has a potent invasive suppressor role.
38.	SFN	Regulates protein synthesis and epithelial cell growth by stimulating Akt/mTOR pathway. Involved in apoptosis and angiogenesis through mTOR pathway.
39.	TPT1	Involved in calcium binding and Regulation of apoptotic process.
40.	ACLY	Involved in lipid synthesis, important drug target as inhibition is known to induce proliferation arrest in cancer cells both in vitro and in vivo, Key Player in Cancer Metabolism, upregulation is reported in various cancers. (ref: DOI: 10.1158/0008-5472.CAN-11-4112)
41.	MMP2	Involved in diverse functions such as remodeling of the vasculature, angiogenesis, tissue repair, tumor invasion, inflammation, and atherosclerotic plaque rupture. Role in ECM degradation, which allows cancer cells to migrate out of the primary tumor to form metastases.
42.	CAV1	Plays a role in promoting cell cycle progression via Ras-ERK pathway. Tumor suppressor gene candidate and a negative regulator of the Ras-p42/44 MAP kinase cascade.
43.	BRCA1	Involved in DNA repair by facilitating cellular responses to DNA damage, act as tumor suppressor. Mutations reported to cause many cancers as defective BRCA1 protein is unable to help fix DNA damages leading to mutations in other genes.
44.	XRCC1	Corrects defective DNA strand-break repair, regulated expression in cancers. Deficient expression of a DNA repair enzyme results in increased un-repaired DNA damages which, through replication errors, lead to mutations and cancer.
45.	NAMPT	Involved in glucose metabolism, housekeeping role in maintaining sugar level in the cell, functions as inhibitor of apoptosis in response to inflammation.
46.	IQGAP1	It associates with calmodulin. Could serve as an assembly scaffold for the organization of a multimolecular complex that would interface incoming signals to the reorganization of the actin cytoskeleton at the plasma membrane. May promote neurite outgrowth. When interacting with PAK6 followed by cascade of signals prevent cells from apoptosis.
47.	GSK3B	GSK-3 activity by phosphorylation by Akt and other kinases has been associated with cancer progression. Also associated with tumor progression by stabilizing components of the beta-catenin complex. (Ref. http://www.ncbi.nlm.nih.gov/pmc/articles/PMC4102778/pdf/oncotarget-05-2881.pdf).
48.	CFL1	Important for normal progress through mitosis and normal cytokinesis. Plays a role in the regulation of cell morphology and cytoskeletal organization. Regulation leads to negative regulation of apoptosis.
49.	DNMT1	”Preferentially methylates hemimethylated DNA that is essential for epigenetic inheritance. Aberrant epigenetic methylation is linked to the onset and progression of cancer. (Ref. Ariel L. Furst, Jacqueline K. Barton DNA Electrochemistry Shows DNMT1 Methyltransferase Hyperactivity in Colorectal Tumors Chemistry & Biology, Volume 22, Issue 7, 23 July 2015, Pages 938-945).”

50.	CTNNB1	Downstream component of the canonical Wnt signaling pathway. Part of a complex of proteins that constitute adherens junctions which are necessary for the creation and maintenance of epithelial cell layers by regulating cell growth and adhesion between cells. Mutations are known to cause various cancers.
51.	RTN4	Signal transduction, signaling with NGF protein responsible for growth regulation of neurons. Involved in apoptosis, tumor suppression, and inhibition of neuronal regeneration. (Ref. Watari A1, Yutsudo M. Apoptosis. 2003 Jan;8(1):5-9. Multi-functional gene ASY/Nogo/RTN-X/RTN4: apoptosis, tumor suppression, and inhibition of neuronal regeneration.)
52.	PHB	Inhibits DNA synthesis. It has a role in regulating proliferation. May play a role in regulating mitochondrial respiration activity and in aging.
53.	CLDN1	Major role in tight junction-specific obliteration of the intercellular space, through calcium- independent cell-adhesion activity. Up-regulation is significantly related to the malignant behavior in several cancer types. Involved in migration and invasiveness of cancer cells. (Ref. Int J Oncol. 2011 Oct;39(4):791-6. doi: 10.3892/ijo.2011.1102. Epub 2011 Jun 28. Expression of CLDN1 in colorectal cancer: a novel marker for prognosis. Nakagawa S1, Miyoshi N, Ishii H, Mimori K, Tanaka F, Sekimoto M, Doki Y, Mori M.).
54.	EP300	Regulates transcription via chromatin remodeling and is important in the processes of cell proliferation and differentiation. Proposed to indirectly increase the transcriptional activity of TP53 through acetylation. Regulation plays a role in dysfunctioning of proper cell growth and division and thus leads to cancerous tumor.
55.	ABCB1	Important protein of the cell membrane that pumps many foreign substances out of cells. It is responsible for decreased drug accumulation in multidrug-resistant cells and often mediates the development of resistance to anticancer drugs.
56.	CTNNA1	Associates with the cytoplasmic domain of a variety of cadherins. Involved in cell-adhesion properties. Also plays a role in tumor metastasis. May play a crucial role in cell differentiation.
57.	STAT1	Member of the Signal Transducers and Activators of Transcription. Mediates cellular responses to interferons (IFNs), cytokine KITLG/SCF and other cytokines and other growth factors. Abnormal activation, due for example to unbalanced signaling or to altered levels promotes cell survival/proliferation, motility. STAT1 is a central mediator of Type I (alpha and beta) and type II (gamma) IFNs, a family of multifunctional secreted proteins involved in cell growth regulation and antiviral and immune defense.
58.	ESR1	The steroid hormones and their receptors are involved in the regulation of eukaryotic gene expression and affect cellular proliferation and differentiation in target tissues. Regulated behavior is found to cause various cancers.

59.	PABPC1	May be involved in cytoplasmic regulatory processes of mRNA metabolism. It plays a role in post-transcriptional control of mRNA and may be involved in tumorigenesis. Reduced expression of PABPC1 accompanies invasive tumors. (Ref. Oncol Rep. 2006 Mar;15(3):667-71. Expression and prognostic roles of PABPC1 in esophageal cancer: correlation with tumor progression and postoperative survival. Takashima N, Ishiguro H, Kuwabara Y, Kimura M, Haruki N, Ando T, Kurehara H, Sugito N, Mori R, Fujii Y.).
60.	FASN	Multi-enzyme protein that catalyzes fatty acid synthesis. The protein codes for FAS and has been investigated as a possible oncogene. In some cancer cell lines, this protein has been found to be fused with estrogen receptor alpha and enhance cellular proliferation and differentiation.
61.	PML	Important in many cellular processes, including tumor suppression, transcriptional regulation, apoptosis, senescence, DNA damage response, and viral defense mechanisms. Acts as the scaffold of PML-NBs allowing other proteins to shuttle in and out, a process, which is regulated by SUMO-mediated modifications and interactions. Positively regulates p53/TP53 by acting at different levels by promoting its acetylation and phosphorylation and by inhibiting its MDM2- dependent degradation. Its expression is cell cycle related and it regulates the p53 response to oncogenic signals.
62.	MTA1	Transcriptional repressor may be involved in the regulation of gene expression by covalent modification of histone proteins. Involved in down-regulation of SIRT1 and thereby is involved in regulation of p53/TP53-dependent apoptotic DNA-damage responses.
63.	PDIA3	Modulate folding of newly synthesized glycoproteins. Downregulation expression is correlated with poor prognosis in early-stage of few cancers. It has also been demonstrated that PDIA3 binds specific DNA fragments in a melanoma cell line. Involved in bone metastasis, which is the most common source of distant relapse in some cancers.

Table S12: **63 cancer common proteins** List of the proteins common to all the cancers considered here with their biological functions.

References

[1] Reed W., Physica A, 319 (2002) 469; Dorogovtsev S. and Mendes J., Proc. R. Soc. London, Ser. B, 268 (2001) 2603; Han D., Qian J. and Ma Y., EPL, 94 (2011) 28006.

[2] Lorimer T., Gomez F. and Stoop R., Sci. Rep., 5 (2015) 12353.

[3] Bierbower S., University of Kentucky Doctoral Dissertations, Paper 778 (2010).

[4] Barabsi AL, Otavi ZN. Network biology: understanding the cells functional organization. Nat Rev Genet. 2004; 5(2):101113. doi: 10.1038/nrg1272 PMID: 14735121.

[5] Ravasz E, Somera AL, Mongru DA, Oltvai ZN, Barabsi AL. Hierarchical Organization of Modularity in Metabolic Networks. Science. 2002; 297(5586):15511555. doi: 10.1126/science.1073374 PMID:12202830.

[6] Albert R, Jeong H, Barabsi AL. Error and attack tolerance of complex networks. Nature. 2000; 406(6794):378382. doi: 10.1038/35019019 PMID: 10935628.

[7] Uhlen, M., Ponten, F., Hober, S., Bergstrom, L., Takanen, J., Nilsson, P., von Feilitzen, K., Lundberg, E. and Navani, S., 2014. Human Protein Atlas. Cell, 7(3), pp.499-508.

[8] Kanehisa, M., Sato, Y., Kawashima, M., Furumichi, M., and Tanabe, M. KEGG as a reference resource for gene and protein annotation. Nuc. Acids Res. 44, D457-D462 (2016).

[9] Kanehisa, M. and Goto, S.; KEGG: Kyoto Encyclopedia of Genes and Genomes. Nuc. Acids Res. 28, 27-30 (2000); Kanehisa, M. The KEGG database. silico simulation of biological processes, 247, pp.91-103 (2002).

[10] Von Mering, C., et al., STRING: known and predicted protein-protein associations, integrated and transferred across organisms. Nucleic Acids Res, 2005. 33(Database issue): p. D433-7.

[11] Gaemers, I.C., et al., A stat-responsive element in the promoter of the episialin/MUC1 gene is involved in its overexpression in carcinoma cells. J Biol Chem, 2001. 276(9): p. 6191-9.

[12] Gao, J., et al., MUC1 is a downstream target of STAT3 and regulates lung cancer cell survival and invasion. Int J Oncol, 2009. 35(2): p. 337-45.

[13] Khodarev, N., et al., Cooperativity of the MUC1 oncoprotein and STAT1 pathway in poor prognosis human breast cancer. Oncogene, 2010. 29(6): p. 920-9.

[14] Kondo, S., et al., MUC1 induced by Epstein-Barr virus latent membrane protein 1 causes dissociation of the cell-matrix interaction and cellular invasiveness via STAT signaling. J Virol, 2007. 81(4): p. 1554-62.

[15] Kufe, D.W., MUC1-C oncoprotein as a target in breast cancer: activation of signaling pathways and therapeutic approaches. Oncogene, 2013. 32(9): p. 1073-81.

[16] Ahmad, R., et al., MUC1-C oncoprotein promotes STAT3 activation in an autoinductive regulatory loop. Sci Signal, 2011. 4(160): p. ra9.

[17] Horm, T.M. and J.A. Schroeder, MUC1 and metastatic cancer: expression, function and therapeutic targeting. Cell Adh Migr, 2013. 7(2): p. 187-98.

[18] Gendler, S.J., MUC1, the renaissance molecule. J Mammary Gland Biol Neoplasia, 2001. 6(3): p. 339-53.

[19] Senapati, S., S. Das, and S.K. Batra, Mucin-interacting proteins: from function to therapeutics. Trends Biochem Sci, 2010. 35(4): p. 236-45.

[20] Papa, L., et al., SOD2 to SOD1 switch in breast cancer. J Biol Chem, 2014. 289(9): p. 5412-6.

[21] Chen, P.M., et al., Activation of NF-kappaB by SOD2 promotes the aggressiveness of lung adenocarcinoma by modulating NKX2-1-mediated IKKbeta expression. Carcinogenesis, 2013. 34(11): p. 2655-63.

[22] Nogueira, V. and N. Hay, Molecular pathways: reactive oxygen species homeostasis in cancer cells and implications for cancer therapy. Clin Cancer Res, 2013. 19(16): p. 4309-14.

[23] Storz, P., Reactive oxygen species in tumor progression. Front Biosci, 2005. 10: p. 1881-96.

[24] Cully, M., et al., Beyond PTEN mutations: the PI3K pathway as an integrator of multiple inputs during tumorigenesis. Nat Rev Cancer, 2006. 6(3): p. 184-92.

[25] Pani, G., O.R. Koch, and T. Galeotti, The p53-p66shc-Manganese Superoxide Dismutase (MnSOD) network: a mitochondrial intrigue to generate reactive oxygen species. Int J Biochem Cell Biol, 2009. 41(5): p. 1002-5.

[26] Peng, S.L., Foxo in the immune system. Oncogene, 2008. 27(16): p. 2337-44.

[27] Taipale, M., D.F. Jarosz, and S. Lindquist, HSP90 at the hub of protein homeostasis: emerging mechanistic insights. Nat Rev Mol Cell Biol, 2010. 11(7): p. 515-28.

[28] Cutress, R.I., et al., BAG-1 expression and function in human cancer. Br J Cancer, 2002. 87(8): p. 834-9.

[29] Held, T., et al., Heat-shock protein HSPA4 is required for progression of spermatogenesis. Reproduction, 2011. 142(1): p. 133-44.

[30] Kang, C.M., et al., Hspa4 (HSP70) is involved in the radioadaptive response: results from mouse splenocytes. Radiat Res, 2002. 157(6): p. 650-5.

[31] Nimmanapalli, R., et al., HSP70 inhibition reverses cell adhesion mediated and acquired drug resistance in multiple myeloma. Br J Haematol, 2008. 142(4): p. 551-61.

[32] Sakurai, T., et al., Heat shock protein A4 controls cell migration and gastric ulcer healing. Dig Dis Sci, 2015. 60(4): p. 850-7.

[33] Wu, C.Y., et al., Induction of HSPA4 and HSPA14 by NBS1 overexpression contributes to NBS1-induced in vitro metastatic and transformation activity. J Biomed Sci, 2011. 18: p. 1.

[34] Meltzer, P.S., Cancer genomics: small RNAs with big impacts. Nature, 2005. 435(7043): p. 745-6.

[35] Visone, R. and C.M. Croce, MiRNAs and cancer. Am J Pathol, 2009. 174(4): p. 1131-8.

[36] Hsu, S.D., et al., miRTarBase: a database curates experimentally validated microRNA-target interactions. Nucleic Acids Res, 2011. 39(Database issue): p. D163-9.

[37] Rajabi, H., et al., MUCIN 1 ONCOPROTEIN EXPRESSION IS SUPPRESSED BY THE miR-125b ONCOMIR. Genes Cancer, 2010. 1(1): p. 62-68.

[38] Sachdeva, M. and Y.Y. Mo, MicroRNA-145 suppresses cell invasion and metastasis by directly targeting mucin 1. Cancer Res, 2010. 70(1): p. 378-87.

[39] Goel, Hira Lal, and Arthur M. Mercurio. ”VEGF targets the tumour cell.” Nature Reviews Cancer 13.12 (2013): 871-882.

[40] Yasuda, Akira, et al. ”Stem cell factor/c-kit receptor signaling enhances the proliferation and invasion of colorectal cancer cells through the PI3K/Akt pathway.” Digestive diseases and sciences 52.9 (2007): 2292-2300.

[41] Claesson Welsh, Lena, and Michael Welsh. ”VEGFA and tumour angiogenesis.” Journal of internal medicine 273.2 (2013): 114-127.

[42] Trinh, Xuan Bich, et al. ”The VEGF pathway and the AKT/mTOR/p70S6K1 signalling pathway in human epithelial ovarian cancer.” British journal of cancer100.6 (2009): 971-978.

[43] Olayioye, Monilola A., et al. ”The ErbB signaling network: receptor heterodimerization in development and cancer.” The EMBO journal 19.13 (2000): 3159-3167.

[44] Mtt, Jorma A., et al. ”Proteolytic cleavage and phosphorylation of a tumor-associated ErbB4 isoform promote ligand-independent survival and cancer cell growth.” Molecular biology of the cell 17.1 (2006): 67-79.

[45] Song, Gang, Gaoliang Ouyang, and Shideng Bao. ”The activation of Akt/PKB signaling pathway and cell survival.” Journal of cellular and molecular medicine9.1 (2005): 59.

[46] Luo, Ji, Brendan D. Manning, and Lewis C. Cantley. ”Targeting the PI3K-Akt pathway in human cancer: rationale and promise.” Cancer cell 4.4 (2003): 257-262.

[47] Pietras, Kristian, et al. ”PDGF receptors as cancer drug targets.” Cancer cell3.5 (2003): 439-443.

[48] Pietras, Kristian, and Arne stman. ”Hallmarks of cancer: interactions with the tumor stroma.” Experimental cell research 316.8 (2010): 1324-1331.

[49] Turner, Nicholas, and Richard Grose. ”Fibroblast growth factor signalling: from development to cancer.” Nature Reviews Cancer 10.2 (2010): 116-129.

[50] Kunii, Kaiko, et al. ”FGFR2-amplified gastric cancer cell lines require FGFR2 and Erbb3 signaling for growth and survival.” Cancer research 68.7 (2008): 2340-2348.

[51] Doll, Laurent, et al. ”Nerve growth factor receptors and signaling in breast cancer.” Current cancer drug targets 4.6 (2004): 463-470.

[52] Vega, Francisco M., and Anne J. Ridley. ”Rho GTPases in cancer cell biology.” FEBS letters 582.14 (2008): 2093-2101.

[53] Sahai, Erik, and Christopher J. Marshall. ”RHOGTPases and cancer.” Nature Reviews Cancer 2.2 (2002): 133-142.

[54] S. L. Braunstein, S. Ghosh & S. Severini The Laplacian of a graph as a density matrix: a basic combinatorial approach to separability of mixed states. Ann. Combinatorics 10, 291317 (2006).

[55] De Domenico, M., Nicosia, V., Arenas, A., and Latora, V. Structural reducibility of multilayer networks. *Nature communications*, **6** (2015).

[56] Barabási AL, Otavi ZN. Network biology: understanding the cells functional organization. Nat Rev Genet. 2004;5(2):101-113.

[57] Ravasz E, Somera AL, Mongru DA, Oltvai ZN, Barabási AL. Hierarchical Organization of Modularity in Metabolic Networks. Science. 2002;297(5586):1551-1555.

Sr.	Pathway	Role of pathway in cancer
1.	Signaling by Vascular endothelial growth factor (VEGF)	VEGF-mediated signalling occurs in tumour cells, and this signalling contributes to key aspects of tumorigenesis, including the function of cancer stem cells and tumour initiation [39].
2.	Signaling by Stem cell factor receptor (SCFKIT)	SCF-kit receptor (KIT) signal transduction on the proliferation and invasion of colorectal cancer cells [40].
3.	VEGFA-VEGFR2 (VEGF family receptor) Pathway	VEGFA signalling acts on tumour cells as a stimulator of the AKT/mTOR pathway as well as as high responses and toxicity in recurrent epithelial ovarian cancer [41, 42].
4.	Signaling by epidermal growth factor receptor 4 (ERBB4)	Signaling by ERBB4 plays important role in tumerogenesis, specifically in tumor development, proliferation and differentiation [43, 44].
5.	Protein kinase B (AKT) signaling	PI3K-Akt pathway is usually genetically selected during tumorigenesis, and the normal cellular functions regulated by this pathway are recruited to promote proliferation and survival of cancer cells [45, 46].
6.	Cellular responses to stress	Individual proteins has their independent role in Cellular responses to stress.
7.	Signaling by Platelet-derived growth factor (PDGF)	PDGF has also been suggested to regulate tumor stroma fibroblasts and tumor angiogenesis and failure in this procedure lead to irregulation [47, 48].
8.	Downstream signaling of activated Fibroblast growth factor receptor 2 (FGFR2)	FGFs and their receptors control a wide range of biological functions, regulating cellular proliferation, survival, migration and differentiation [49, 50].
9.	Signaling by Nerve Growth Factor (NGF)	Survival and proliferation of cancer cells are strongly stimulated by NGFs. Also having role neuronal cell differentiation [51].
10.	Signaling by Rho GTPases	The members of this pathway are involved in GTP hydrolysis and involve in the acquisition of a motile and invasive phenotype. Rho GTPases have been found to contribute to pathological processes including cancer cell migration, invasion, and metastasis, inflammation, and wound repair [52, 53].
11.	Axon guidance	Individual proteins has their independent role in Axon guidance in cancer.
12.	Innate Immune System	Individual proteins has their independent role in Innate Immune System in cancer.
13.	Signal Transduction	Individual proteins has their independent role in Signal Transduction in cancer.
14.	Metabolism of proteins	Individual proteins has their independent role in Metabolism of proteins in cancer.

Table S7: **Role of pathways in cancer**

Sr.	Name of protein	Functions in cancer
1.	Mucin 1, cell surface associated	Mucins are O-glycosylated proteins that play an essential role in forming protective mucous barriers on epithelial surfaces. These proteins also play a role in intracellular signaling. Overexpression, aberrant intracellular localization, and changes in glycosylation of this protein have been associated with carcinomas.
2.	Superoxide dismutase 2, mitochondrial	Mutations in this gene have been associated with idiopathic cardiomyopathy (IDC), premature aging, sporadic motor neuron disease, and cancer.
3.	Heat shock 70kDa protein 4 (HspA4)	Cancer-related genes, Plasma proteins, Predicted intracellular proteins. Widely distributed cytoplasmic expression, nuclear expression to a lesser extent.
4.	Heat shock 70kDa protein 5 (HspA5)	Disease related genes, Plasma proteins, Predicted secreted proteins. The protein encoded by this gene is a member of the heat shock protein 70 (HSP70) family. It is localized in the lumen of the endoplasmic reticulum (ER), and is involved in the folding and assembly of proteins in the ER. As this protein interacts with many ER proteins, it may play a key role in monitoring protein transport through the cell.
5.	MAPK1	Elevated and constitutive activation of ERK1/2 has been detected in a large number of human tumors; including colon, kidney, gastric, prostate, breast, non-small cell lung cancer, bladder, chondrosarcomas and glioblastoma multiforme which show especially high frequencies of kinase activation. The reason for constitutive activation of the ERK pathway in the majority of tumor cells seems to be due to a disorder in RAF, RAS, EGFR or other upstream signaling molecules. In addition, several studies have shown that the ERK-MAPK pathway can directly promote cell motility and the migration of tumor cells.
6.	STAT1	Signal transducer and activator of transcription 1,In response to cytokines and growth factors, STAT family members are phosphorylated by the receptor associated kinases, and then form homo- or heterodimers that translocate to the cell nucleus where they act as transcription activators. This protein can be activated by various ligands including interferon-alpha, interferon-gamma, EGF, PDGF and IL6. This protein mediates the expression of a variety of genes, which is thought to be important for cell viability in response to different cell stimuli and pathogens.

Table S8: **Functions of 6 proteins in cancer. (Source: The Human Protein Atlas and Gene Cards)**

Sr.	Protein of Interest	miRNAs regulating	Status in cancer	Significance
1.	MUC1	miR-145	DR in lung, bladder, colorectal and esophageal cancer	Promotes survival
		miR-1226		
		miR-335	DR in breast cancer	Promotes metastasis
		miR-125	DR in lung, esophageal, epithelial ovarian, and bladder cancer UR in prostate, breast and liver cancer	DR promotes invasion/metastasis UR promotes metastasis by inhibiting Bak1
		miR-124	DR in colorectal cancer	Promotes invasion/metastasis
		miR-21	UR in lung, breast, liver, bladder, prostate, esophageal and epithelial ovarian cancer	Promotes survival, metastasis and therapy resistance by inhibiting PTEN, BMPRII, RECK, TIMP3, BCL2, PDCD4, TPMI.
		miR-455		
2	SOD2	miR-222	UR in lung, breast and prostate cancer	Promotes resistance to apoptosis by inhibiting PTEN
		miR-377	NA	
		miR-17	DR in prostate cancer	Promotes migration/invasion
		miR-30	DR in breast and bladder cancer	Promotes metastasis
		miR-769	NA	
		miR-342	NA	
		miR-4325	NA	
		miR-212	NA	
3	HSPA4	miR-1	DR in lung cancer	Promotes tumorigenesis
		miR-30	DR in breast and bladder cancer	Promotes resistance to apoptosis by inhibiting PTEN
		miR-26	UR in colorectal cancer	Promotes invasion/metastasis by inhibiting PTEN
		miR-16	NA	
4	HSPA5	miR-199	DR in epithelial ovarian cancer	Promotes angiogenesis and inflammation
		miR-30	DR in breast and bladder cancer	Promotes resistance to apoptosis by inhibiting PTEN
		miR-335	DR in breast cancer	Promotes metastasis
		miR-122	NA	
		miR-16	NA	
		miR-93	NA	

Table S9: **Status and functions of miRNAs regulating the Group ”A” proteins in cancers (Source: KEGG)**

γ	$\gamma_N > \gamma_D$	$\gamma_N < \gamma_D$	$\gamma_N = \gamma_D$
γ^k	Ovarian	Oral	Breast, Cervical, Lung, Colon, Prostate
γ^{CC}	NA	Breast, Oral, Cervical, Colon	Ovarian, Lung, Prostate
γ_λ^L	Breast	Oral	Ovarian, Cervical, Lung, Colon, Prostate

Table S11: The table depicts the γ corresponding to various entropies where γ_N is the entropy for normal dataset and γ_D corresponds to the entropy of disease dataset.
